# Supramolecular Architectures of Nucleic Acid/Peptide Hybrids

**DOI:** 10.3390/ijms21249458

**Published:** 2020-12-12

**Authors:** Sayuri L. Higashi, Normazida Rozi, Sharina Abu Hanifah, Masato Ikeda

**Affiliations:** 1 United Graduate School of Drug Discovery and Medical Information Sciences, Gifu University, 1-1 Yanagido, Gifu 501-1193, Japan; s.higgashi@gmail.com; 2 Department of Chemical Sciences, Faculty of Science and Technology, Universiti Kebangsaan Malaysia, Bangi 43600, Selangor, Malaysia; adizam_92@yahoo.com (N.R.); sharina@ukm.edu.my (S.A.H.); 3 Department of Chemistry and Biomolecular Science, Faculty of Engineering, Gifu University, 1-1 Yanagido, Gifu 501-1193, Japan; 4 Center for Highly Advanced Integration of Nano and Life Sciences, Gifu University, 1-1 Yanagido, Gifu 501-1193, Japan; 5 Institute for Glyco-Core Research (iGCORE), Gifu University, Furo-cho, Chikusa-ku, Nagoya 464-8603, Japan

**Keywords:** supramolecular architectures, self-assembly, nanostructures, nucleic acids, peptides, molecular hybrid

## Abstract

Supramolecular architectures that are built artificially from biomolecules, such as nucleic acids or peptides, with structural hierarchical orders ranging from the molecular to nano-scales have attracted increased attention in molecular science research fields. The engineering of nanostructures with such biomolecule-based supramolecular architectures could offer an opportunity for the development of biocompatible supramolecular (nano)materials. In this review, we highlighted a variety of supramolecular architectures that were assembled from both nucleic acids and peptides through the non-covalent interactions between them or the covalently conjugated molecular hybrids between them.

## 1. Introduction

**Figure 1:**
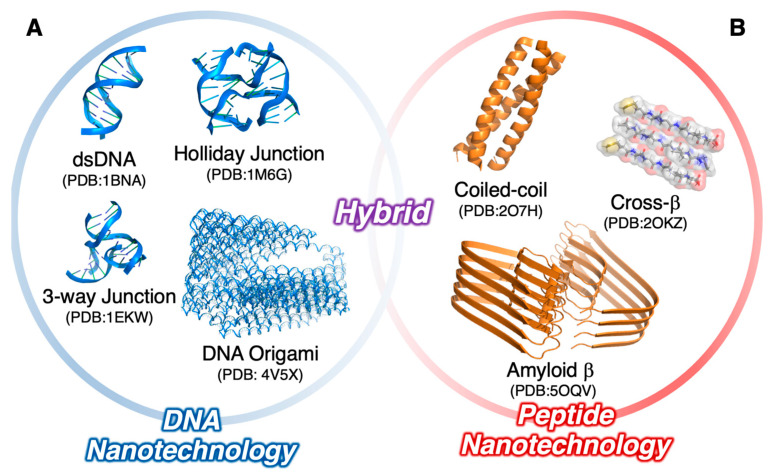
(**A**) DNA and (**B**) peptide nanotechnologies. Representative supramolecular architectures (nanostructures) and their typical component structural motifs are shown.

The construction of supramolecular architectures comprising nucleic acids as well as peptides (including oligopeptides and polypeptides in this paper) has greatly progressed recently [1,2,3]. Particularly, deoxyribonucleic acid (DNA) nanotechnology, a concept that was introduced by Seeman in the 1980s [4,5], has greatly expanded the range of rationally designed nanostructures (Figure 1A). For instance, the DNA origami technology, which was invented by Rothemund [6,7], is widely accepted as a promising strategy to construct supramolecular architectures with custom nanoscale shapes via the bottom-up approach. Therein, a long single-stranded DNA (ssDNA) was folded by the addition of hundreds of short ssDNAs, namely, staple strands, followed by one-pot thermal annealing to obtain the rationally designed shape of the supramolecular architectures (the DNA origami). Apart from the DNA origami technology, the DNA tiles and bricks (in which the long ssDNAs are not prerequisite but the sequence-designed ssDNAs with the characteristic structural motif like the holiday junction [8,9,10,11] are required) offer supramolecular architectures with various morphology such as fibers [12,13,14], tubes [15,16,17,18,19,20], and polyhedrons [21,22,23]. Evidently, the high fidelity of the predictable Watson–Crick (WC) base pairings and the regular formation of the well-defined double-stranded DNA (dsDNA) structures are indispensable to the design and construction of such beautiful supramolecular architectures based on DNA nanotechnology.

These DNA nanostructures offer a variety of bio-applications, such as scaffolds for enzymes in the construction of bioreactors [24,25], vehicles for drug delivery [26], biosensors [27], and robotics [28,29,30,31]. Despite these promising functions and advantages of DNA nanostructures, several drawbacks are directly related to the intrinsic properties of DNA. One of the most fundamental drawbacks is the relatively low stability of DNA against nucleases that exist rather abundantly under physiological conditions or in biological fluids [32,33]. Furthermore, several DNA nanostructures require high salt concentrations, which are occasionally incompatible with biosystems and/or deviate significantly from the standard salt concentration for biological fluids or cell culture media [34]. However, to overcome these drawbacks, a variety of strategies, including molecular hybrid approaches that are described herein, has been proposed and investigated [35,36,37,38,39,40].

The self-assembly of oligopeptides or polypeptides is also an actively investigated research topic in the field of biomolecular science (Figure 1B) [41,42,43]. For example, the self-assembled fibrous nanostructures of proteins, such as actin filaments and microtubules, are keys to cellular movement and elasticity [44]. Such fibrous self-assembled nanostructures of peptides are present both inside and outside living cells [45]. Particularly, amyloid fibril formations are beneficial research targets because of their severe pathological outcomes [46]. Very recently, the detailed structure of the amyloid-β peptide in the potentially pathological self-assembled state was successfully solved at the atom level by cryogenic transmission electron microscopy (cryoTEM) techniques [47]. As shown there, most of the amyloid fibril comprised the β-sheet structures, that is, the cross-β structures. In the cross-β structural motif, the peptide strands folded into (at least partially) elongated β-strands to self-assemble into (or stack) fibrous structures in which the long axis of the peptide strands was perpendicular to that of the fibrous structures. Conversely, very recently, self-assembled fibrous nanostructures comprising an α-helix-folded peptide (the cross-α structures) were observed for the first time [48,49]. This unique finding and the other structural motifs of the peptide self-assemblies under investigation, such as the coiled-coil structures [50,51,52], could further elucidate the design of supramolecular peptide architectures.

The above briefly mentioned supramolecular architectures that are composed of nucleic acids or peptides could show several advantages in the research fields of supramolecular architectures. First of all, biomolecules bear potential biocompatibility and biodegradability as well as they are versatile and sustainable. In addition, reliable automatic synthesis for nucleic acids and peptides have been established [53,54,55,56] so that those with designated length and sequence can be synthesized and are even commercially available. These well-established synthetic technologies have allowed for the progress of the research fields.

Despite the rapid progress in the development of supramolecular architectures that are composed solely of nucleic acids or peptides, the combination of the two biomolecules for the development of hybrid supramolecular architectures remains less explored. Therefore, this review non-exhaustively aims to highlight recently published examples to underscore hierarchical structures and the promising (future) applications of supramolecular architectures that comprised both nucleic acids and peptides by dividing them into two sections: Section 2 (nucleic acid/oligopeptide hybrids) and Section 3 (nucleic acid/polypeptide (protein) hybrids), based on their component peptides, oligopeptides (generally <~20 amino acids (aa) and peptides that are obtained by solid-phase peptide synthesis without ligations or gene expression (recombinant) technology) or polypeptides (proteins), by referring to “peptides with ˂~10–20 residues as oligopeptides and those with more residues as polypeptides” [57].

## 2. Nucleic Acid/Oligopeptide Hybrids

### 2.1. Non-Covalently Conjugated Nucleic Acid/Oligopeptide Hybrids

In this subsection, we introduce supramolecular architectures that are constructed from nucleic acids and oligopeptides (without any covalent conjugation between them) in which the non-covalent bond interactions between them are in turn indispensable. On the basis of the final morphology (spherical, fibrous, etc.) of the constructed supramolecular architectures, we further discussed them in several subsubsections (Section 2.1.1, Section 2.1.2, and Section 2.1.3). In most of the examples, the oligopeptides bearing nucleic acid-binding domains derived from nucleic acid-binding proteins or simple cationic peptide domains were fused to self-assembling peptides with or without spacers to obtain the constituent oligopeptide derivatives. The inherent structures, e.g., one-dimensional and circular, of nucleic acids and/or their supramolecular nanostructures are used as templates for directing the formation of hybrid supramolecular architectures. Consequently, the sizes, shapes, and stoichiometric compositions of the final hybrid supramolecular architectures are difficult to predict. Nevertheless, rather monodispersed and uniformly fabricated supramolecular architectures have been occasionally constructed by exploiting the recent progress in DNA and peptide nanotechnology. In the following subsubsections, we highlighted some recent but not comprehensive reports [24] on such well-defined supramolecular architectures.

#### 2.1.1. Spherical Supramolecular Architectures

**Figure 2:**
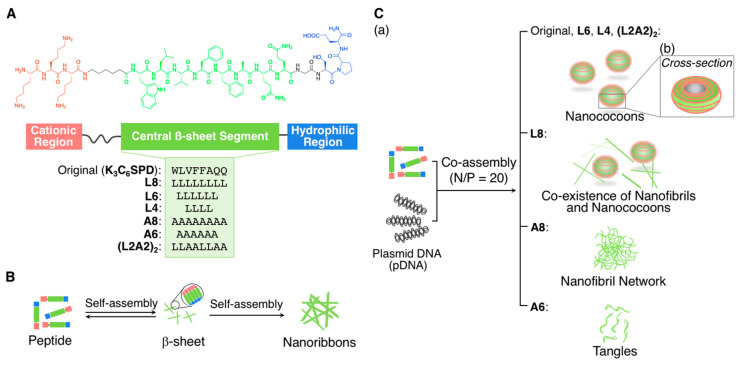
(**A**) Chemical structure of the self-assembling peptide derivatives containing **K_3_**, a self-assembling β-sheet domain, and a hydrophilic segment at the C-terminus for controlled water dispersibility. (**B**) Schematic illustration of the self-assembly of the peptide derivatives to form the nanoribbons. (**C**) (**a**) Schematic illustration of the co-assembly of the peptide derivatives with plasmid DNA (pDNA) to form a variety of hybrid nanostructures. (**b**) Cross-sectional view of the nanococoons. Adapted from [58]. Copyright 2014 American Chemical Society. Adapted from [59]. Copyright 2017 John Wiley and Sons Publisher.

A series of well-organized spherical supramolecular architectures of nucleic acids and oligopeptides were recently developed by Ni and Chau [58,59,60]. For instance, they developed a parapoxvirus mimetic ellipsoid, that is, “nanococoons” (approximately 65 × 45 nm^2^, as estimated by atomic force microscopy (AFM) and TEM), by the synergistic co-assembly of a self-assembling peptide derivative (**K_3_C_6_SPD**: KKK-C6-WLVFFAQQ-GSPD, 15 aa, and a C6 spacer; Figure 2A) and a plasmid DNA (pDNA, 4.7 kbp) [58]. The **K_3_C_6_SPD** peptide contains a DNA-binding cationic tripeptide domain (K_3_), a self-assembled β-sheet domain (a hydrophobic segment that was derived from an amyloid-β peptide {Aβ (17–21)}), and a hydrophilic segment at the C-terminus, which was designed according to viral capsid proteins, for controlled water dispersibility. As illustrated in Figure 2B, **K_3_C_6_SPD** can independently self-assemble into antiparallel β-sheet bilayer-based filamentous nanoribbons (approximately 4 × 12 nm^2^, as estimated by AFM and TEM). Upon adding pDNA in an appropriate n/p ratio of 20, **K_3_C_6_SPD** was simultaneously coated around pDNA and eventually formed the nanococoons (Figure 2C(a)). The unique structural model of the nanococoons was proposed, as shown in Figure 2C(b), based on the TEM images and the other analyses. Noteworthily, the nanococoons exhibited significant tolerance against degradations by enzymes, e.g., trypsin or chymotrypsin as the proteases and deoxyribonuclease I (DNase I) as the nucleases, most probably because of the strong association between the self-assembled **K_3_C_6_SPD** and pDNA and the compactly assembled total structure.

Thereafter, the same research group successfully tuned the morphology, stability, and cellular uptake (gene transfection) efficiency of the peptide–DNA virus-mimicking complexes by modulating the amino acid sequence in the central self-assembling β-sheet segment (originally, 8 aa: WLVFFAQQ) of the peptide components [59]. Exhaustively, six different central segments that comprised leucine (L) and/or alanine (A) (**L8**: LLLLLLLL, **L6**: LLLLLL, **L4**: LLLL, **A8**: AAAAAAAA, A6: AAAAAA, and (**L2A2**)_2_: LLAALLAA, from 8 to 4 aa) were investigated (Figure 2A), and it was observed that increasing the hydrophobicity of the amino acid side chains (from A to L) and the length (from 4 to 8 aa) of the central segment of the peptides enhanced the inter-nanofibril association and thus controlled the final morphology of the peptide/DNA virus-mimicking complexes. Various morphologies, e.g., the tangles for **A6**/DNA, the nanofibril networks for **A8**/DNA, the striped nanococoons for **L4** or **L6** or (**L2A2**)_2_/DNA, and the coexistence of nanofibrils and nanococoons for **L8**/DNA, were observed in the n/p ratio of 20 (Figure 2C(a)). Expectedly, the strength of the inter-nanofibril association regulated the stability of the structure and DNA protection, as well as the gene transfection efficiency of the nanococoons. Fortunately, such a strong inter-nanofibril association that increased the stability of the nanococoon structures did not exert significantly negative impact on the gene transfection efficiency in their study, and the strategy might be beneficial for exploring gene delivery systems.

**Figure 3:**
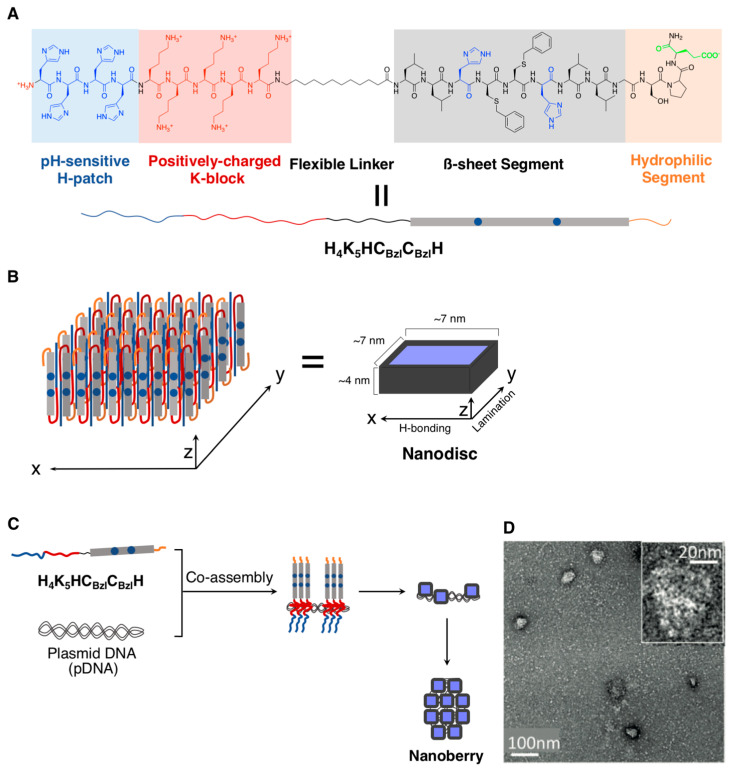
(**A**) Chemical structure of the self-assembling peptide derivative, **H_4_K_5_HC_Bzl_C_Bzl_H**. (**B**) Schematic illustration of a nanodisc that was constructed via the self-assembly of H_4_K_5_HC_Bzl_C_Bzl_H. (**C**) Schematic illustration and (**D**) representative TEM image (magnified in inset) of the co-assembly between **H_4_K_5_HC_Bzl_C_Bzl_H** with pDNA to form the nanoberries. Adapted from [60]. Copyright 2020 John Wiley and Sons Publisher.

Very recently, the same research group constructed pH-responsive peptide/DNA complexes, that is, “nanoberries,” with a structurally lower aspect ratio (a more planer structure) [60] than that of the nanococoons described above. These nanoberries comprised a peptide (**H_4_K_5_HC_Bzl_C_Bzl_H**: HHHHKKKKK-C12-LLHC_Bzl_C_Bzl_HLLGSPD; 21 aa, including an unnatural amino acid, “benzylated cysteine (C_Bzl_),” and a C12 spacer; Figure 3A), and pDNA (4.7 kbp), as shown in Figure 3B. The **H_4_K_5_HC_Bzl_C_Bzl_H** peptide was rationally designed from similar peptides and optimized to install pH responsiveness through the introductions of histidine residues at the DNA-binding domain, as well as the self-assembling central domain (to promote endosomal escape inside living cells) and the aromatic residues of C_Bzl_ for the formation of the self-assembled nanostructure. Indeed, **H_4_K_5_HC_Bzl_C_Bzl_H** was assembled into uniform nanodiscs (approximately 7 × 7 nm^2^ (width × length), as estimated by TEM, and approximately 4 nm (height), as estimated by AFM) comprising β-sheet structures. Upon complexation with pDNA, the peptide/DNA complexes, which exhibited the morphology of the nanoberries (approximately 50 nm, as estimated by TEM), were obtained (Figure 3C,D); a virus-mimetic structural model of the nanoberries, in which the core DNA was wrapped by nanodiscs, was proposed. Crucially, the nanoberries exhibited a distinct structural transition from the compactly packed nanoberries to loosen complexes with heterogeneous sizes due to the acidic pH shift (from 7 to 5). Subsequently, the encapsulated DNA was released via polyanion exchange with heparin (an anionic polysaccharide). Such an acidic pH shift-initiated stepwise structural changes and the concurrent release of the encapsulated DNA for the virus-mimicking artificial peptide/DNA nanoberries could greatly contribute to gene delivery like natural viruses. Indeed, the transfection ability of the nanoberries was demonstrated by an enhanced expression of the green fluorescent protein (GFP) that was encoded into the encapsulated pDNA.

#### 2.1.2. Fibrous Supramolecular Architectures

**Figure 4:**
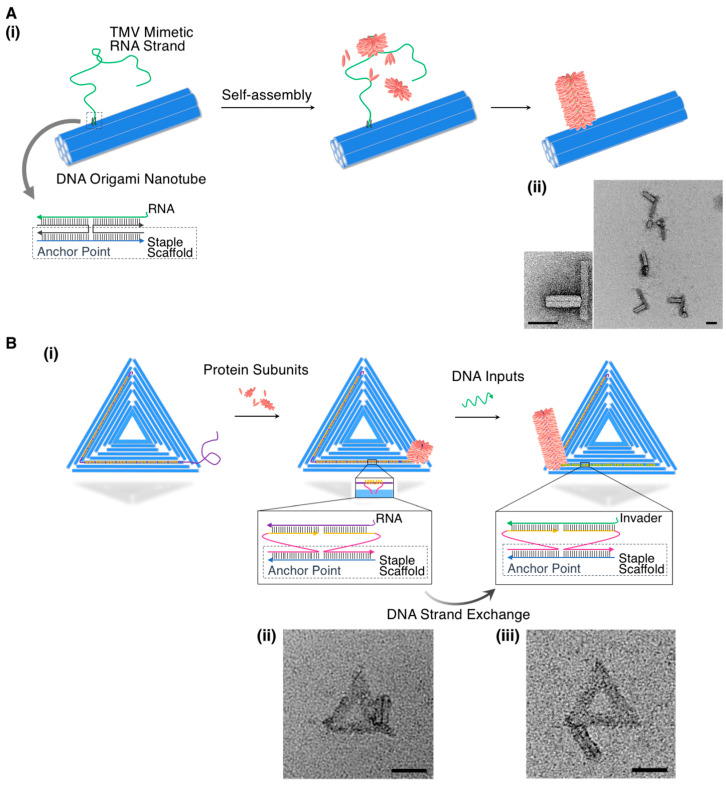
(**A**) (**i**) Schematic illustration of the formation of a semi-artificial tobacco mosaic virus (TMV) nanostructure at a designated position of the DNA origami nanotube and (**ii**) representative TEM images of the in situ assembled protein nanotubes on DNA origami nanotubes with one docking site. (**B**) (**i**) Schematic illustration of the spatiotemporally controlled TMV protein assembly on the surface of a DNA origami triangle, (**ii**,**iii**) representative TEM images of each state illustrated in (**i**). Scale bar: 50 nm. Adapted from [64,65]. Copyright 2018 and 2020 American Chemical Society.

Since the early stage of supramolecular chemistry and self-assembly [61,62], fibrous tobacco mosaic virus (TMV) has been one of the most attractive and referenced fibrous bio-supramolecular architectures comprising a single-stranded genetic ribonucleic acid (ssRNA) (approximately 6400 nt) that is surrounded by constituent coat proteins [63]. Very recently, the bio-supramolecular architecture of TMV was applied to construct morphologically unique supramolecular architectures by combining it with the DNA origami nanotechnology. For example, Wang and coworkers recently designed a DNA origami nanotube that was tethered to an ssRNA strand via hybridization with a complementary DNA strand that was connected to the nanotube [64]. The in situ assembly of the TMV coat proteins formed semi-artificial TMV nanostructures at designated positions of the DNA origami nanotubes, as shown in Figure 4A. More recently, they successfully controlled the spatiotemporally precise assembly of the TMV protein more on the surface of a DNA origami triangle in a similar design [65], although they applied a toehold mediated strand displacement (TMSD) reaction, as outlined in Figure 4B. In detail, the short DNA strands that protruded from the DNA origami triangle were designed to bind a specific sequence of the ssRNA strand, which was captured at programmed points on the surface of the triangle. The programmed points functioned as “locks” to stop the assembly of the TMV protein. Thereafter, a complementary short DNA strand containing a toehold region released ssRNA via the TMSD reaction, thereby resulting in the TMV protein assembly up to the following “locks” point. This strategy allowed the precise control of a specific protein assembly within the kinetic system containing a travel pathway, the positions, and the assembly stoichiometries, which was crucial to the construction of further sophisticated and functional hybrid nanostructures.

**Figure 5:**
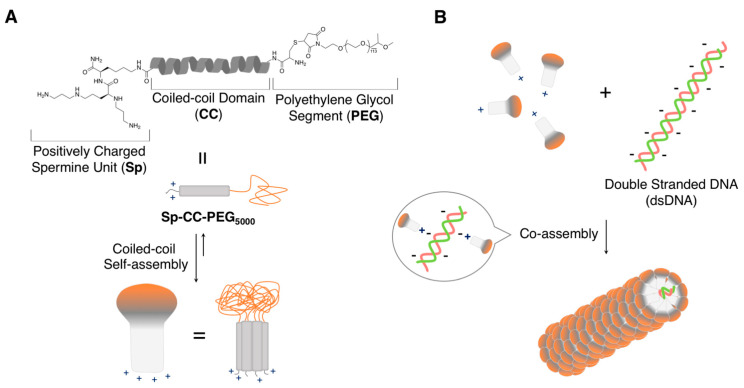
(**A**) Design of SP-CC-PEG to form mushroom-shaped nanostructures. (**B**) Schematic illustration of the formation of supramolecular rod-like nanostructures through the complexation of SP-CC-PEG_5000_ with dsDNAs. Adapted from [66]. Copyright 2013 American Chemical Society.

To artificially create a virus-like filamentous supramolecular architecture with a well-defined length, Stupp and coworkers described a distinct strategy, which applies the self-assembly of the designed artificial peptide-based nanostructures (Figure 5) on a linear or circular dsDNA, as a template [66]. This sophisticated supramolecular design would be a reminiscence of the aforementioned TMVs of biosystems. The mushroom-shaped nanostructures were constructed through the self-assembly of triblock peptide-based molecules (PEGylated coiled-coil peptides: **SP-CC-PEG**) containing a cationic spermine unit at one terminus (a binding site for dsDNA); a coiled-coil peptide (REGVAKALRAVANALHYNASALEEVADALQKVKM: 34 aa), which aggregates into heptameric structures; and a long poly(ethylene glycol) (PEG) chain, which imparts it with water solubility, as well as steric repulsion between the PEG clusters in the self-assembled structure. Interestingly, the longer PEG was critical to both the formation of the monodispersed filamentous complexes and the suppression of amorphous (not-well-ordered structure) formation. Upon complexing **SP-CC-PEG_5000_** with dsDNAs (150, 300, 600, and 1200 bp) or extended supercoiled pDNAs (2.7, 4.4, and 10.2 kbp), homogenous supramolecular rod-like objects with controlled lengths were successfully constructed. The development of this kind of supramolecular architectures in which the nucleic acids are enwrapped in the peptides and/or their supramolecular nanostructures have been actively investigated for applications in non-viral gene delivery systems [67].

**Figure 6:**
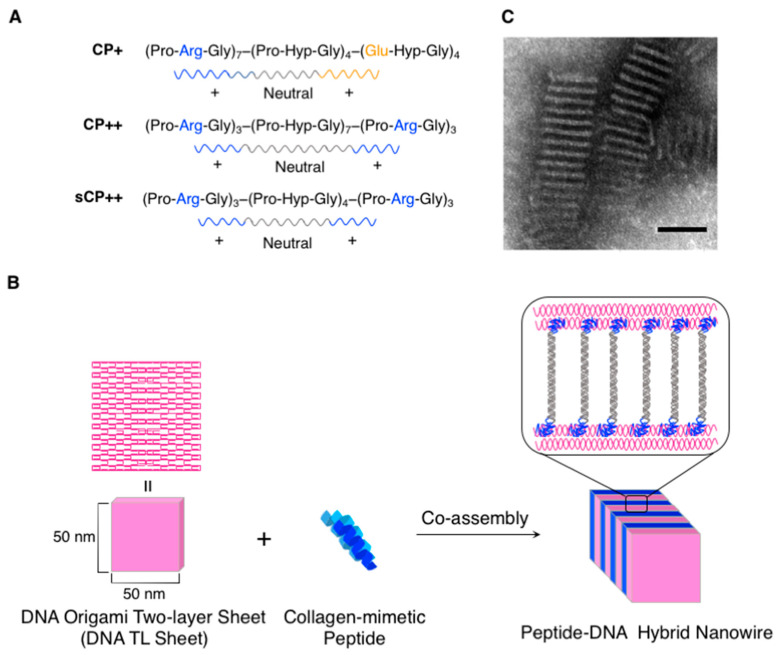
(**A**) Sequences of the three collagen-like triple helix-forming peptides (**CP+**, **CP++**, and **sCP++**). (**B**) Schematic illustration of the co-assembly of the two-layered DNA origami nanosheets (DNA TL nanosheets) and the collagen-mimetic peptides (**CP++** and **sCP++**) to obtain the one-dimensional peptide/DNA hybrid nanowires. (**C**) Representative TEM images of assembled with DNA TL nanosheets and **CP++**. Scale bar: 50 nm. Adapted from [68]. Copyright 2017 American Chemical Society.

Ke and coworkers have demonstrated that the co-assembly of two-layered DNA origami nanosheets (DNA TL nanosheets) and collagen-mimetic peptides obtained one-dimensional peptide/DNA hybrid nanowires (Figure 6) [68]. Specifically, three collagen-like triple helix-forming peptides (**CP+**: (PRG)_7_(P-Hyp-G)_4_(E-Hyp-G)_4_ {45 aa}, **CP++**: (PRG)_3_(P-Hyp-G)_7_(PRG)_3_ {39 aa}, and **sCP++**: (PRG)_3_(P-Hyp-G)_4_(PRG)_3_ {30 aa}; Figure 6A) were investigated for the construction of well-defined hybrid nanostructures with the DNA TL nanosheets. It would be reasonable to expect that the polyanionic DNA TL nanosheets (approximately 50 × 50 × 5 nm^3^, estimated by TEM) would form complexes with the cationic collagen-like triple helices bearing arginine-rich overhangs through electrostatic attractions. They initially confirmed that the arrangement of **CP+** on the DNA TL nanosheet was not structurally well-ordered. In sharp contrast, **CP++** and **sCP++** bearing cationic (PRG) triads in both the N- and C-terminal ends co-assembled with the DNA TL nanosheets to form striped well-ordered nanowire structures. Crucially, the distances between the TL nanosheets (the inter-sheet distances) were successfully estimated by TEM and synchrotron small- and wide-angle X-ray scattering (SAXS/WAXS) and were well consistent with the length of the cationic peptides. This indicated that the peptide helices could align perpendicularly to the surface of the DNA TL nanosheets, as depicted in Figure 6B,C.

**Figure 7:**
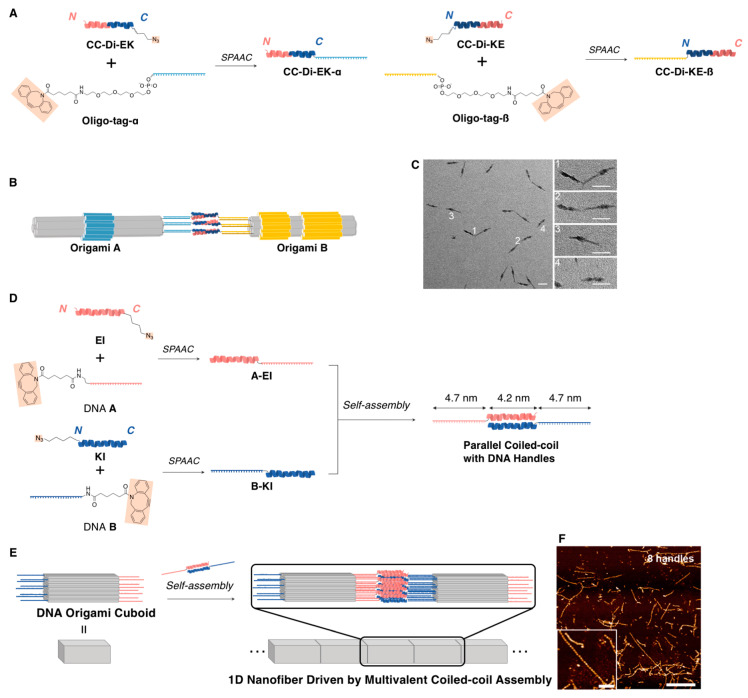
(**A**) Sequences of the coiled-coil heterodimers that formed the peptides (**CC-Di-EK** and **CC-Di-KE**) that were conjugated to the oligonucleotides (**α**, **β**). (**B**) Schematic illustration and (**C**) a representative TEM image of the connection of two distinct one-dimensional DNA origami nanostructures (**Origami**s **A** and **B**) by the specific formation of peptide coiled-coil heterodimers. (**D**) Sequences of the coiled-coil heterodimers that formed the peptides (**EI** and **KI**) that were conjugated to the oligonucleotides (DNA **A** and **B**). (**E**) Schematic illustration and (**F**) representative AFM images to obtain long one-dimensional arrays of DNA origami cuboid nanostructures. Scale bar: 50 nm for **C** and 1 μm (250 nm (inset)) for **F**. Adapted from [69,70]. Copyright 2019 and 2020 American Chemical Society.

The two-dimensional, as well as the one-dimensional DNA origami nanostructures, were organized by exploiting specific peptide self-assemblies. For instance, Turberfield and coworkers have very recently reported that two distinct one-dimensional DNA origami nanostructures can be connected by the specific formation of peptide coiled-coil heterodimers (**CC-Di-EK**: Ac-GEIAALEQ-ENAALEQ-KIAALKW-KNAALKQG: 30 aa, **CC-Di-KE**: Ac-GKIAALKQ-KNAALKY-EIAALEQ-ENAALEQG: 30 aa), as depicted in Figure 7A. The overall charges of each peptide were designed to be close to or exactly zero at a neutral pH to avoid nonspecific electrostatic interactions. To optimize the formation of the connected heterodimer structure as depicted in Figure 7B, the number of peptides (*n*) to be attached to one side of each one-dimensional DNA origami nanostructure was varied from 1 to 3. The TEM observations clearly revealed the formation of the heterodimer, and the formation efficiency attained approximately 40% at *n* = 3, although it was <5% at *n* = 1 or 2 (Figure 7C) [69]. Moreover, further elongated nanostructures (micrometer-long one-dimensional arrays) were constructed by a similar architectural design to that of Stephanopoulos and coworkers. They designed similar peptide coiled-coil heterodimers (**EI**: Ac-EIAALEK-EIAALEK-ENAALEW-EIAALEK: 28 aa, **KI**: Ac-KIAALKE-KIAALKE-KNAALKW-KIAALKE: 28 aa (Figure 7D)), although they attached the two distinct peptides to both sides of the DNA origami cuboid nanostructure (16 × 19.5 × 32 nm^3^) through DNA–DNA hybridizations, which allowed for the formation of an elongated one-dimensional array, as shown in Figure 7E. They also investigated the optimal number of peptides (*m*) required for the design and observed via AFM that the formation of long arrays comprising 30–45 cuboids occurred at *m* = 8 (Figure 7F). Such long arrays were not observed at *m* = 10 and 12, and shorter oligomers (mainly ˂15 cuboids) were mainly obtained at *m* < 8 (0, 1, 2, 4, and 6). This clearly indicated that *m* = 8 would be optimal to obtain long one-dimensional arrays of the DNA origami cuboid nanostructure for reasons that remain currently unclear according to their study [70].

#### 2.1.3. Orthogonal Supramolecular Architectures Without Random Co-Assembly

**Figure 8:**
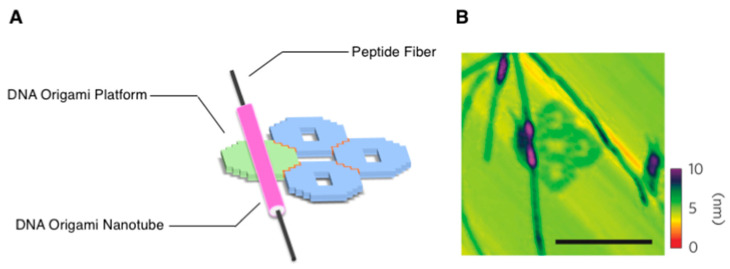
(**A**) Schematic illustration and (**B**) a representative AFM image of amyloid fibril-filled DNA origami nanotubes that were organized onto two-dimensional DNA origami platforms. Scale bars: 250 nm. The colour scale bar indicates height. Adapted from [75]. Copyright 2014 Springer Nature.

Orthogonal supramolecular architectures through self-sorting phenomena to form plural distinct supramolecular nanostructures of molecular constituents without a random co-assembly have attracted increased attention [71,72,73,74]. In this context, Seeman and coworkers developed DNA origami nanotubes in which the amyloid fibrils were sheathed, and the resultant fibril-filled DNA origami nanotubes were further organized into the other DNA origami platforms (two-dimensional planar monolayer structures that comprised four flat DNA origami hollow square motifs) via DNA–DNA hybridizations, as depicted in Figure 8A [75]. To construct the self-sorted, fibril-filled DNA origami nanotubes, a staple DNA strand (31 nt) was covalently conjugated to a fibril-forming peptide (AcTTR1-GGK {TTR_105–115_ with a tripeptide (GGK) segment extension and an acetyl cap at the N-terminus}; Ac-YTIAALLSPYSGGK: 14 aa) that was derived from the amyloidogenic protein transthyretin (TTR). The staple DNA that conjugated with AcTTR1-GGK afforded a nucleation site, which was positioned inside the DNA origami nanotube. Notably, the negatively charged fibril-forming sequence (AcTTR1-GGE; Ac-YTIAALLSPYSGGE: 14 aa) was designed to avoid the nonspecific interactions between the DNA nanotubes and the peptide fibrils. The fibril formation of the AcTTR1-GGE peptide without DNA, which indicated the presence of a typical cross-β structure, was first revealed by TEM and AFM observations, as well as X-ray fiber diffraction. After the thermal annealing of an M13 scaffold DNA strand with staple strands including one, which was conjugated to AcTTR1-GGK, the formation of an unbranched amyloid fibril proceeded simultaneously through the addition of the AcTTR1-GGE peptides. The formation of the fiber inside the DNA origami nanotubes was indirectly demonstrated by several constructs, e.g., the decoration of gold nanoparticles on the outer surface of the DNA origami nanotubes. Finally, a programmable DNA design could enable the precise positioning of fibril-filled DNA origami nanotubes on prepared two-dimensional planar DNA origami platforms, which was further demonstrated by AFM (Figure 8B).

**Figure 9:**
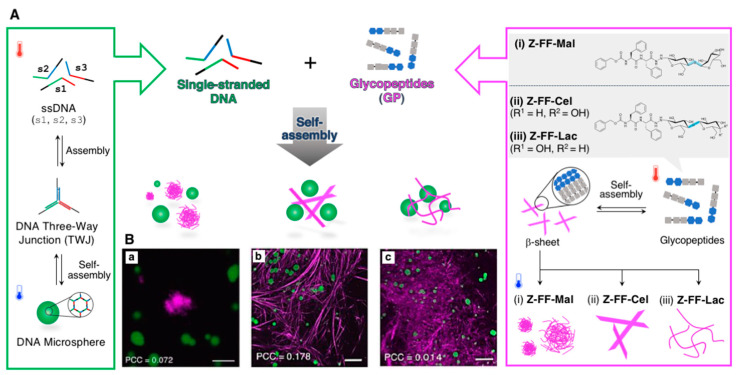
(**A**) Schematic illustration of the orthogonal self-assembly of semi-artificial glycopeptides (right panel) and ssDNAs (left panel) to produce hybrid soft materials that comprised supramolecular nanostructures of glycopeptide and DNA microspheres. (**B**) Representative confocal laser scanning microscopy (CLSM) images showing the orthogonal coexistence of DNA microsphere and the supramolecular nanostructures of (**a**) **Z-FF-Mal**, (**b**) **Z-FF-Cel**, and (**c**) **Z-FF-Lac**. Scale bar: 5 µm for **a** and 10 µm for **b**,**c**. Adapted from [76]. Copyright 2019 John Wiley and Sons Publisher.

Very recently, we revealed the orthogonal coexistence of DNA microspheres, which can be obtained from three ssDNAs (30 nt), and supramolecular nanostructures (helical nanofibers, straight nanoribbons, and flowerlike microaggregates) of semi-artificial glycopeptides comprising a diphenylalanine (FF) dipeptide (vide infra), as shown in Figure 9 [76]. One of the main factors responsible for such orthogonal assembly (self-sorting) that was induced only by simple one-pot and one-step thermal annealing, would be the distinct driving forces of the self-assembly, i.e., the specific WC base pairings of the DNA strands and the cross β-sheet assembly of the glycopeptides. Additionally, the selective degradation propensity (in response to biostimuli, such as a protease, DNase, or ssDNA) of each supramolecular nanostructure was retained and could enable the construction of active soft nanomaterials with excellent biofunctions. We believe that the combination of self-assembling nucleic acids and peptides would be a promising strategy for constructing such orthogonal supramolecular architectures, as described in this review, thus enabling the construction of more complex and functional hybrid supramolecular architectures that are otherwise unachievable and yet inaccessible [77].

### 2.2. Covalently Conjugated Nucleic Acid-Oligopeptide Hybrids

In this subsection, we mainly highlighted supramolecular architectures comprising covalently conjugated nucleic acid–oligopeptide hybrids as their components. This subsection is further divided into two subsubsections (Section 2.2.1: spherical, Section 2.2.2: fibrous) based on the primary morphology of the supramolecular architectures. Generally, covalent conjugations cause the self-assembly of the resultant molecular hybrids into supramolecular architectures more predictably when either the nucleic acid or oligopeptide component exhibits robust ability to form specific supramolecular architectures. Indeed, most of the following recent research exploited the conjugation of self-assembling peptides onto the nucleic acids of interest, thereby producing novel supramolecular architectures under given aqueous conditions.

#### 2.2.1. Spherical Supramolecular Architectures

**Figure 10:**
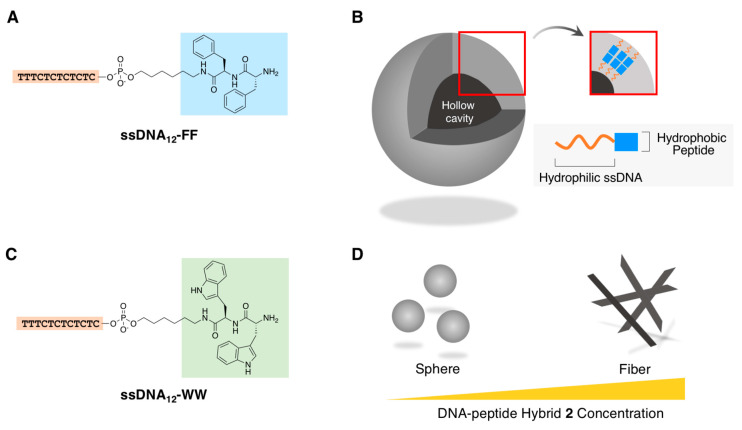
(**A**) Chemical structure of **ssDNA_12_-FF**. (**B**) Plausible supramolecular architectures of the hollow vesicular structures that were obtained through the self-assembly of **ssDNA_12_-FF**. (**C**) Chemical structure of **ssDNA_12_-WW**. (**D**) Plausible, concentration-dependent supramolecular architectures of **ssDNA_12_-WW** obtained by its self-assembly. Adapted from [91,92]. Copyright 2012 and 2014 Royal Chemical Society.

Since the discovery of self-assembly ability of the FF dipeptide to form nanotubes in water [78], it has been used as a promising motif to design self-assembling molecules [79,80,81,82,83,84,85,86,87,88,89,90]. Thus, the design of a covalently conjugated hybrid containing the FF dipeptide and an oligo DNA (ssDNA: 5′-CTCTCTCTCTTT-3′, 12 nt), that is, **ssDNA_12_-FF** (Figure 10A), was expected, followed by an investigation of the formations of their corresponding supramolecular architectures [91]. Initial assessments by Vebert-Nardin and coworkers via microscopic observations (AFM and scanning electron microscopy) revealed that spherical supramolecular nanostructures with sizes ranging from 200 to 300 nm were successfully obtained by the direct dissolution of **ssDNA_12_-FF** in water (2 mg/mL), followed by extrusion through a filter membrane (0.45 µm). Such a morphology was not observed by only mixing the unmodified FF dipeptide with the component oligo DNA (12 nt) without covalent conjugation. Furthermore, their hollow vesicular structures (Figure 10B) were made evident by detailed microscopic observations (TEM and confocal microscopy) and the encapsulation ability of a hydrophilic fluorescence dye. Subsequently, the same research group reported that another aromatic dipeptide (the tryptophan (WW) dipeptide), which conjugated with the same oligo DNA (ssDNA: 5′-CTCTCTCTCTTT-3′, 12 nt), **ssDNA_12_-WW** (Figure 10C), yielded a spherical supramolecular nanostructure with a size similar to that of its FF counterpart (described above) at a lowered concentration. Interestingly, at an increased concentration, e.g., 1.0 mM, **ssDNA_12_-WW** produced fibrous supramolecular nanostructures (the diameter ranged between 0.5 and 1.0 µm and the length was several micrometers), as depicted in Figure 10D [92].

**Figure 11:**
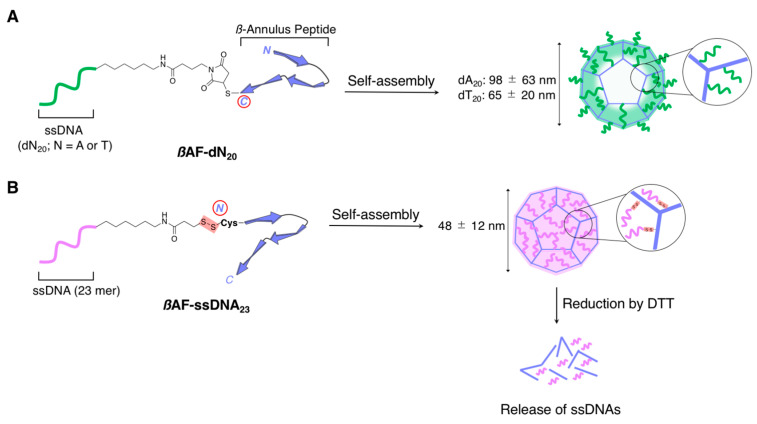
(**A**) Schematic representation of the self-assembly of β-annulus fragment (**βAF**)_dN_20_ [N = A (**βAF_dA_20_**) or T (**βAF_dT_20_**)] to form spherical capsular architectures that displayed the nucleic acids at their exterior surfaces. (**B**) Schematic representation of the self-assembly of **βAF_ssDNA_23_** to form spherical capsular architectures that encapsulated the nucleic acids. Adapted from ref 88. Copyright 2017 John Wiley and Sons Publisher. Adapted from [95]. Copyright 2019 the Chemical Society of Japan.

Spherical capsular architectures, which displayed nucleic acids at their surfaces (exterior), were successfully constructed by Matsuura and coworkers [93] through the self-assembly of a DNA/peptide hybrid, **βAF-dA_20_** (Figure 11A), comprising a peptide (INHVGGTGGAIMAPVAVTRQLVGS, 24 aa) that was covalently conjugated to the nucleic acid (ssDNA: A20, 20 nt). The 24-mer peptide is a β-annulus fragment (**βAF**), which is a structural motif of the internal skeleton of the tomato bushy stunt virus capsid. The research group demonstrated that the 24-mer β-annulus peptide fragment could self-assemble into spherical capsular nanostructures with a size of 30–50 nm in water [94]. Similarly, **βAF-dA_20_** could self-assemble into spherical nanostructures, as depicted in Figure 11A, which was observed by TEM and dynamic light scattering (DLS) measurements (98 ± 63 nm, the average hydrodynamic diameter). Although the DNA strand (dA_20_) was attached to the 24-mer β-annulus peptide fragment, **βAF-dA_20_** could still self-assemble at lower concentrations compared with that of the original DNA-unmodified peptide fragment. Similarly, the spherical nanostructures obtained from **βAF-dT_20_** (Figure 11A) with an average hydrodynamic diameter of 65 ± 20 nm (estimated by DLS) were constructed under the same conditions. Thereafter, to confirm the hybridization ability of DNAs that were displayed on the spherical nanostructures, the two spherical nanostructures were mixed equimolarly. The TEM observations and DLS measurements revealed the presence of their aggregates, which were constructed through the hybridization of complementary DNAs (dA_20_•dT_20_). Further experimental pieces of evidence for the DNA hybridization ability of the spherical nanostructures of **βAF-dT_20_** were obtained by complexing **βAF-dT_20_** with long complementary DNA strands. For example, the addition of polydA caused extensive aggregations of the **βAF-dT_20_** spherical nanostructures, whereas no aggregation was observed upon adding polydT. Subsequently, another DNA/peptide hybrid, **βAF-ssDNA_23_** (ssDNA_23_: TCTACAAAGGGAAGCCCTTTCTG; 23 nt) in which the ssDNA strands were directed inside the capsular architectures, was designed (Figure 11B). The obtained DNA-encapsulated spherical nanostructure exhibited reduction-responsive disassembly that was due to the reductive cleavage of the disulfide bonds between DNA and the peptide, which eventually resulted in the controlled release of the encapsulated DNAs [95]. Overall, the spherical architectures of the 24-mer peptide β-annulus conjugates were sufficiently robust [96,97,98,99,100,101,102,103] so that various DNAs could be introduced into the exterior and interior parts of the capsular structure since the introduced DNA did not destroy the ability of the peptides to form the capsular structure.

**Figure 12:**
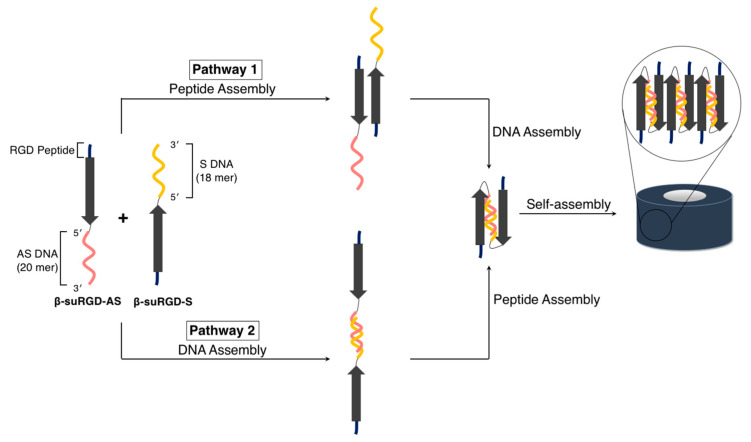
Sequences of the two DNA/peptide hybrids, **β-suRGD-AS** and **β-suRGD-S**, and the two distinct protocols that controlled their assembly pathway to yield the monodispersed toroidal nanostructures (**β-suRGD-AS**/**β-suRGD-S**). Adapted from [104]. Copyright 2016 John Wiley and Sons Publisher.

Lim and a coworker designed two DNA/peptide hybrids, **β-suRGD-AS** and **β-suRGD-S** (Figure 12), comprising ssDNAs (**β-suRGD-AS**: GCGAGCTGCACGCTGCCGTC, 20 nt; **β-suRGD-S**: GACGGCAGCGTGCAGCTC, 18 nt) and a β-sheet peptide (**β-suRGD**: KWKWEWYWKWEWKRGDRGD, 19 aa) containing a self-assembly motif and a cationic segment. They demonstrated that the simultaneous and orthogonal molecular self-assembly abilities of DNA and the peptide moiety in the hybrids (**β-suRGD-AS** and **β-suRGD-S**) enabled the construction of well-defined toroidal nanostructures. More specifically, the two DNA/peptide hybrids (**β-suRGD-AS** and **β-suRGD-S**) were mixed in a 1:1 stoichiometric ratio, whereas two distinct protocols were applied to control the assembly pathways (pathway 1: peptide assembly to DNA assembly, pathway 2: DNA assembly to peptide assembly), as outlined in Figure 12. The two pathways caused the formation of toroidal nanostructures, as revealed by cryoTEM (3D reconstruction of a single particle) (particle size = 9 nm, as estimated by TEM, AFM, and DLS), which were almost identical (**β-suRGD-AS**/**β-suRGD-S**). By contrast, heterogeneous structures were obtained by simple dissolution (without any protocol to control the assembly pathways) most probably because of the formations of kinetically trapped structures [104,105]. Interestingly, the structural transition from the monodispersed toroidal nanostructures to the entangled fibers, including the giant toroidal structures, could be induced upon adding mRNA, which contains a sequence (20 nt) that is complementary to DNA **AS**, and this should be related to the antisense effect of the nanostructures (**β-suRGD-AS**/**β-suRGD-S**) demonstrated in the study.

#### 2.2.2. Fibrous Supramolecular Architectures

**Figure 13:**
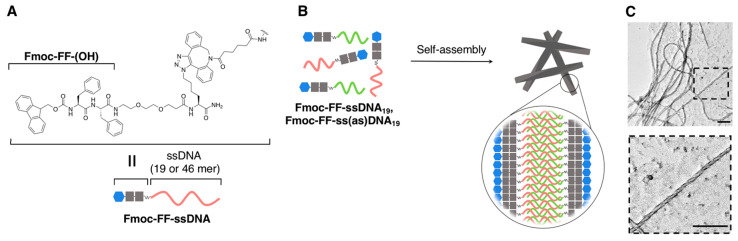
(**A**) Chemical structure of **Fmoc-FF-ssDNA**s. (**B**) Schematic representation of the self-assembly of **Fmoc-FF-ssDNA**s to form hierarchical nanostructures through DNA hybridization. (**C**) Representative TEM images obtained from co-assembly of **Fmoc-FF-ssDNA_19_** and **Fmoc-FF-ss(as)DNA_19_**. Scale bar: 200 nm. Adapted from [109]. Copyright 2019 American Chemical Society.

As described by several examples in Section 2.2.1, the morphology of supramolecular architectures that are obtained from covalently conjugated nucleic acid–oligopeptide hybrids is conditional. Fibrous supramolecular nanostructures could be preferentially obtained by strengthening the self-assembly ability of peptides for the direct formation of one-dimensional structures. Indeed, fibrous supramolecular nanostructures have been constructed more efficiently by attaching ssDNA to fluorenylmethyloxycarbonyl **(Fmoc)-FF**, as shown in Figure 13A. The Fmoc group (one of the most common protecting groups for amino acids) is currently widely accepted as a powerful self-assembly motif because of its ability to form one-dimensional supramolecular nanostructures [106,107,108]. Interestingly, Freeman and coworkers revealed that the morphology of the fibrous supramolecular nanostructures strongly depended on the length of ssDNA [109]. In detail, **Fmoc-FF-ssDNA_19_** (ssDNA_19_: CTCAGTGGACAGCCTTTTT; 19 nt) afforded twisted fibers with average width and pitch of 20 and 150 nm, respectively, whereas **Fmoc-FF-ssDNA_46_** (ssDNA_46_: CAGTACAGTTTCGTCCAACGCTCCAGAACTGAGGCTGTCCACTGAG: 46 nt) afforded loosely twisted but wider fibers compared with those of **Fmoc-FF-ssDNA_19_** with average width and pitch of 44 and 630 nm, respectively. Under the investigated conditions, **Fmoc-FF** did not produce twisted fibers, thus suggesting that helical structures and their propensity to form self-assembled structures of **Fmoc-FF-ssDNA**s are dictated by the length of ssDNA. Additionally, they confirmed that the hybridization of the DNA segment in **Fmoc-FF-ssDNA_46_** caused fiber bundling, which could be more clearly observed in the co-assembly of **Fmoc-FF-ssDNA_19_** and **Fmoc-FF-ss(as)DNA_19_** in which the complementary ssDNA (ss(as)DNA_19_) against ssDNA_19_ was introduced, as shown in Figure 13B,C. Accordingly, precise the base pairing (hybridization) ability of nucleic acids (DNA) was undoubtedly beneficial to the control of assembled structures at molecular and supramolecular levels, as highlighted in this paper. In fact, Stupp and coworkers, including the same author (Freeman), reported earlier that peptide amphiphiles, which contained nucleic acids, produced super-structured networks with controlled and reversible bundling of supramolecular fibrous structures [110]. In the study, more crucially, the bundling of the fibrous structures influenced the phenotypic transformations in the astrocytes (neural cells) that were in contact with the materials. Conversely, the polyanionic property of nucleic acid can, in turn, afford a means of controlling the nucleation of the peptide self-assembly, which might be involved in polyanion-induced amyloid fibrillation that is associated with the corresponding diseases. For example, Lyn and coworkers recently succeeded to obtain high-resolution structural information on the self-assembled nanostructures of a positively charged peptide (Ac-KLVIIAG-NH_2_: 7 aa) that was templated by nucleic acids, which was facilitated by the structural complementarity between the nucleic acid backbone and the antiparallel cross-β structures of the peptides for electrostatic interactions [111].

## 3. Nucleic Acid/Polypeptide (Protein) Hybrids

In this section, we briefly highlighted supramolecular architectures comprising nucleic acid/polypeptide(protein) hybrids as their components, and the hybrids are discussed in two subsections based on their types of conjugations.

### 3.1. Non-Covalently Conjugated Hybrids

**Figure 14:**
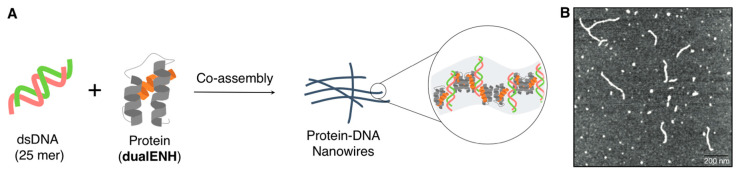
(**A**) Schematic representation of the formation of nanowires comprising a computationally designed protein/**dualENH** and a short dsDNA. (**B**) A representative AFM image obtained after mixing **dualENH** with the dsDNA (25 bp). Adapted from [112]. Copyright 2014 Springer Nature.

Mayo and coworkers designed and constructed well-defined supramolecular nanowires comprising dsDNA/artificially modified protein (**dualENH**) complexes in which a sophisticated supramolecular assembly was obtained via selective protein–protein interaction (homodimerization) that was aided by a computational design, as well as by inherent protein/dsDNA interactions [112]. They selected the well-studied *Drosophila* engrailed homeodomain (ENH) as the scaffold protein. Homeodomains, including ENH, are common eukaryotic DNA-binding domains comprising three helices (conventionally defined as 60 aa in length based on homology) [113]. The homodimerization interface of **dualENH** was engineered on the exterior sides of helices 1 and 2 of ENH that are structurally opposite its DNA-binding helix 3. To obtain a one-dimensional DNA/**dualENH** assembly, dsDNA requires two protein-binding sites with the desired geometry (180°). Specifically, upon complexing dsDNA (25 bp) containing an 11-nucleotide binding motif (TAATTTAATTT, TAATTT: ENH-binding motif) with **dualENH**, the nanowire structures (width, ~15 nm; length, ~300 nm; as estimated by AFM) were successfully obtained. Noteworthily, the crystal structure of the complex was also solved in which a slightly kinked (may not reflect the structure of the nanowire in the solution state) but infinitely repeated formation of the dsDNA/protein nanowires was evident (Figure 14).

### 3.2. Covalently Conjugated Hybrids

#### 3.2.1. Linear Supramolecular Architectures

**Figure 15:**
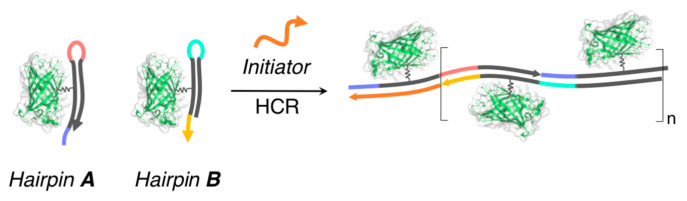
Schematic representation of the formation of linear supramolecular architectures that tethered the proteins by exploiting hybridization chain reaction (HCR) of nucleic acids. Adapted from [116]. Copyright 2018 American Chemical Society.

Recently, the hybridization chain reaction (HCR) of nucleic acids is recognized as a robust strategy for constructing dsDNA-based supramolecular architectures for a variety of bio-applications [114,115]. As a covalently conjugated DNA/protein hybrid, Mirkin and coworkers constructed a set of mutant GFPs bearing a hairpin-shaped ssDNA and demonstrated the formation of linear supramolecular oligomers (several tens of nanometers in length, as estimated by AFM), which tethered the protein through a chain growth polymerization reaction that was induced by the addition of an initiator (ssDNA) [116] (Figure 15). Notably, a further extension of the chain could be induced because the HCR system exhibited a living character that was similar to the living polymerization of covalent polymers. Furthermore, the same research group very recently reported that the length of the linear supramolecular oligomers can be finely modulated by carefully designing a metastable hairpin-shaped ssDNA (with the introduction of mismatched base pairs) that would be attached to the protein [117].

#### 3.2.2. Cage-Like Supramolecular Architectures

**Figure 16:**
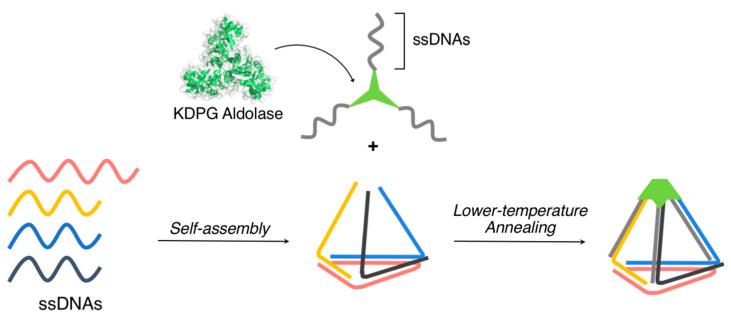
Three-dimensional cage-like supramolecular architecture obtained by the complexation of a homotrimeric protein containing three ssDNAs with a triangular DNA nanostructure bearing three complementary ssDNAs through DNA hybridization. Adapted from [118]. Copyright 2019 American Chemical Society.

Stephanopoulos and coworkers developed discrete three-dimensional cage-like supramolecular architectures by exploiting the specific self-assembly process of both the proteins and the nucleic acids [118]. A homotrimeric protein containing three ssDNAs was constructed through the covalent conjugation of ssDNAs to a cysteine residue (introduced by site-directed mutagenesis) of 2-dehydro-3-deoxyphosphogluconate aldolase (25 kDa, *C*_3_-symmetric homotrimer) (Figure 16). This covalently conjugated DNA/protein hybrid can form a discrete three-dimensional cage-like supramolecular architecture by complexing with a triangular DNA nanostructure bearing three complementary ssDNAs through DNA hybridization (Figure 16). The size of the three-dimensional cage-like supramolecular architecture can be varied by changing the lengths of DNAs. Indeed, two different sizes of the supramolecular architectures (triangular pyramid: 8 nm (height) × 10 nm (side of the triangle), 12 nm (height) × 14 nm (side of the triangle)) were constructed and visualized via AFM.

#### 3.2.3. Crystalline Supramolecular Architectures

**Figure 17:**
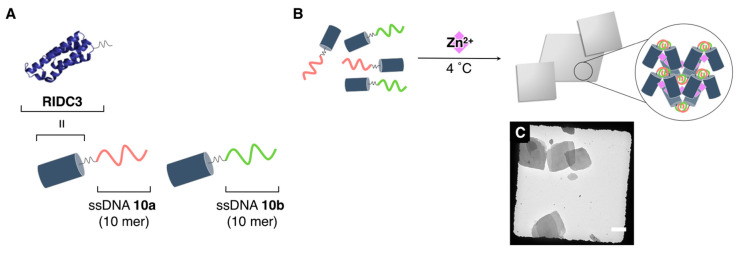
(**A**) Molecular design of the nucleoproteins (**RIDC3-10a** and **RIDC3-10b**). (**B**) Schematic representation and (**C**) a representative TEM image of the ordered crystalline architectures obtained through the complexation of **RIDC3-10a**/**10b** with Zn^2+^ under a small window of conditions. Scale bar: 5 µm. Adapted from [119]. Copyright 2018 American Chemical Society.

Tezcan and coworkers designed and constructed discrete crystalline nucleoprotein architectures through the three distinct cooperative interactions: (i) the WC base pairing, (ii) the DNA/protein interactions, and (iii) the protein–metal coordination [119]. Figure 17 shows the molecular design of the nucleoprotein (covalently conjugated protein/nucleic acid hybrid). Therein, a modified cytochrome, **RIDC3** (an engineered variant of the monomeric four-helix bundle protein cytochrome, *cb*_562_), which was developed by the same group [120,121,122], was used as the protein component. Complementary ssDNAs (TTATTAAAA and TTTTAATTAA for **RIDC3-10a** and **RIDC3-10b**, respectively, 10 nt) were attached to **RIDC3** by exploiting a single cysteine residue of **RIDC3**. Essentially, **RIDC3** itself can self-assemble into one-, two-, and three-dimensional crystalline arrays through Zn^2+^-mediated interactions (protein–metal coordination). As shown in Figure 17, ordered crystalline architectures (thin, micrometer-sized crystals) were obtained from **RIDC3-10a** and **RIDC3-10b**, although under a rather small window of conditions {pH (4.75–5), temperature (4–10 °C), and stoichiometry between Zn^2+^ and **RIDC3-10a**/**10b** (2–10 equiv.)}. For example, the solutions of **RIDC3-10a**/**10b** did not produce any discretely assembled architecture (crystals) in the absence of Zn^2+^. Furthermore, the addition of ethylenediaminetetraacetic acid to the suspension of the **RIDC3-10a**/**10b** crystals dissolved it, as well as its incubation at >40 °C. These results implied that Zn^2+^- and DNA hybridization-mediated interactions are keys to the formation of the ordered crystalline architectures. Furthermore, by combining several techniques (negative stain TEM, cryoTEM, SAXS, and molecular dynamics simulations), the structural characterization of the ordered crystalline architectures was comprehensively accomplished.

## 4. Conclusions

One challenge for this research field is the hierarchical combination of plural supramolecular architectures obtained from not only self-assembling peptides but also self-assembling nucleic acids at meso-scale as described in Section 2.1.3. Such examples are still rare and often require a multi-step and tedious process. Nevertheless, careful molecular design enabling the orthogonal molecular self-assembling could allow for simple one-pot production process to obtain unique hierarchical supramolecular architectures [76,77]. Another simple but important challenge is cost issue. Although reliable and reproducible automatic synthesis of nucleic acids and peptides are available, the production cost issue remains, especially for DNA origami requiring over a hundred ssDNAs with different length and sequence [123] Biotechnological mass production might be optional, whereas it poses additional issues such as sterilization and batch-to-batch variability, which could result in the increase in the production costs. It might be worth noting that catalytic peptide synthesis has attracted increasing attentions recently [124,125,126,127,128], which could overcome the production cost issue of peptides.

In a natural system, the ribosome, which consists of RNA and polypeptides, i.e., ribosomal RNA (rRNA) and proteins, respectively [129,130,131,132], is one of the most complex hierarchical bio-supramolecular architectures and exhibits a precise function to synthesize proteins (decoding center or factory). This hybrid bio-supramolecular architecture clearly reflects a strong structure–function relationship through the construction of evolutional but sophisticated hybrid architectures. In sharp contrast, the formations of rather structureless liquid droplets in living cells, namely, the liquid–liquid phase separation, have very recently attracted increased attention in the fundamental and applied research fields of biology, as well as chemistry [133,134,135]. Recent studies have revealed that the dynamic and transient formation of nucleic acid/protein droplets in the cells is closely related to crucial biological processes, such as cell polarization [136,137,138,139]. These findings could significantly affect the design principles of molecular hybrid materials in the future.

Conclusively, this review has described a variety of supramolecular architectures that were assembled from both nucleic acids and peptides with structural orders that ranged from the molecular to nano-scales. The rational and modular molecular and structural designs for the construction and engineering of such supramolecular architectures to equip them with desired functions and properties would facilitate the elucidation of their beneficial bio-applications, such as sensor [140], cell-culturing matrix for regenerative medicine [141,142,143], and drug-releasing material [144,145,146,147]. Furthermore, synergistically combining the strengths of both molecules (nucleic acids and peptides) would be essential to widening the scope of future research projects.

## References

[1] Seeman N.C., Sleiman H.F. (2018). DNA nanotechnology. Nat. Rev. Mater..

[2] Madsen M., Gothelf K.V. (2019). Chemistries for DNA nanotechnology. Chem. Rev..

[3] Heuer-Jungemann A., Liedl T. (2019). From DNA tiles to functional DNA materials. Trends Chem..

[4] Seeman N.C. (2003). DNA in a material world. Nature.

[5] Seeman N.C. (1982). Nucleic acid junctions and lattices. J. Theor. Biol..

[6] Rothemund P.W.K. (2006). Folding DNA to create nanoscale shapes and patterns. Nature.

[7] Hong F., Zhang F., Liu Y., Yan H. (2017). DNA origami: Scaffolds for creating higher order structures. Chem. Rev..

[8] Kallenbach N.R., Ma R.-I., Seeman N.C. (1983). An immobile nucleic acid junction constructed from oligonucleotides. Nature.

[9] Fu T.J., Seeman N.C. (1993). DNA double-crossover molecules. Biochemistry.

[10] Wang Y., Mueller J.E., Kemper B., Seeman N.C. (1991). Assembly and characterization of five-arm and six-arm DNA branched junctions. Biochemistry.

[11] Wang X., Chandrasekaran A.R., Shen Z., Ohayon Y.P., Wang T., Kizer M.E., Sha R., Mao C., Yan H., Zhang X. (2018). Paranemic crossover DNA: There and back again. Chem. Rev..

[12] Winfree E., Liu F., Wenzler L.A., Seeman N.C. (1998). Design and self-assembly of two-dimensional DNA crystals. Nature.

[13] Yan H. (2003). DNA-templated self-assembly of protein arrays and highly conductive nanowires. Science.

[14] Schulman R., Winfree E. (2007). Synthesis of crystals with a programmable kinetic barrier to nucleation. Proc. Natl. Acad. Sci. USA.

[15] Rothemund P.W.K., Ekani-Nkodo A., Papadakis N., Kumar A., Fygenson D.K., Winfree E. (2004). Design and characterization of programmable DNA nanotubes. J. Am. Chem. Soc..

[16] Yin P., Hariadi R.F., Sahu S., Choi H.M.T., Park S.H., LaBean T.H., Reif J.H. (2008). Programming DNA tube circumferences. Science.

[17] Liu H., Chen Y., He Y., Ribbe A.E., Mao C. (2006). Approaching the limit: Can one DNA oligonucleotide assemble into large nanostructures?. Angew. Chem. Int. Ed..

[18] Wilner O.I., Orbach R., Henning A., Teller C., Yehezkeli O., Mertig M., Harries D., Willner I. (2011). Self-assembly of DNA nanotubes with controllable diameters. Nat. Commun..

[19] Zhang D.Y., Hariadi R.F., Choi H.M.T., Winfree E. (2013). Integrating DNA strand-displacement circuitry with DNA tile self-assembly. Nat. Commun..

[20] Liu X., Zhao Y., Liu P., Wang L., Lin J., Fan C. (2019). Biomimetic DNA nanotubes: Nanoscale channel design and applications. Angew. Chem. Int. Ed..

[21] He Y., Ye T., Su M., Zhang C., Ribbe A.E., Jiang W., Mao C. (2008). Hierarchical self-assembly of DNA into symmetric supramolecular polyhedra. Nature.

[22] Zhang C., He Y., Su M., Ko S.H., Ye T., Leng Y., Sun X., Ribbe A.E., Jiang W., Mao C. (2009). DNA self-assembly: From 2D to 3D. Faraday Discuss..

[23] Tian C., Li X., Liu Z., Jiang W., Wang G., Mao C. (2014). Directed self-assembly of DNA tiles into complex nanocages. Angew. Chem. Int. Ed..

[24] Fu J., Yang Y.R., Johnson-Buck A., Liu M., Liu Y., Walter N.G., Woodbury N.W., Yan H. (2014). Multi-enzyme complexes on DNA scaffolds capable of substrate channelling with an artificial swinging arm. Nat. Nanotechnol..

[25] Linko V., Eerikäinen M., Kostiainen M.A. (2015). A modular DNA origami-based enzyme cascade nanoreactor. Chem. Commun..

[26] Yu Y., Jin B., Li Y., Deng Z. (2019). Stimuli-responsive DNA self-assembly: From principles to applications. Chem. Eur. J..

[27] Xiao M., Lai W., Man T., Chang B., Li L., Chandrasekaran A.R., Pei H. (2019). Rationally engineered nucleic acid architectures for biosensing applications. Chem. Rev..

[28] Douglas S.M., Bachelet I., Church G.M. (2012). A logic-gated nanorobot for targeted transport of molecular payloads. Science.

[29] Li S., Jiang Q., Liu S., Zhang Y., Tian Y., Song C., Wang J., Zou Y., Anderson G.J., Han J.-Y. (2018). A DNA nanorobot functions as a cancer therapeutic in response to a molecular trigger in vivo. Nat. Biotechnol..

[30] Kopperger E., List J., Madhira S., Rothfischer F., Lamb D.C., Simmel F.C. (2018). A self-assembled nanoscale robotic arm controlled by electric fields. Science.

[31] Nummelin S., Shen B., Piskunen P., Liu Q., Kostiainen M.A., Linko V. (2020). Robotic DNA nanostructures. ACS Synth. Biol..

[32] Mei Q., Wei X., Su F., Liu Y., Youngbull C., Johnson R., Lindsay S., Yan H., Meldrum D. (2011). Stability of DNA origami nanoarrays in cell lysate. Nano Lett..

[33] Hahn J., Wickham S.F.J., Shih W.M., Perrault S.D. (2014). Addressing the instability of DNA nanostructures in tissue culture. ACS Nano.

[34] Kielar C., Xin Y., Shen B., Kostiainen M.A., Grundmeier G., Linko V., Keller A. (2018). On the stability of DNA origami nanostructures in low-magnesium buffers. Angew. Chem. Int. Ed..

[35] Mikkilä J., Eskelinen A.-P., Niemelä E.H., Linko V., Frilander M.J., Törmä P., Kostiainen M.A. (2014). Virus-encapsulated DNA origami nanostructures for cellular delivery. Nano Lett..

[36] Auvinen H., Zhang H., Nonappa, Kopilow A., Niemelä E.H., Nummelin S., Correia A., Santos H.A., Linko V., Kostiainen M.A. (2017). Protein coating of DNA nanostructures for enhanced stability and immunocompatibility. Adv. Healthc. Mater..

[37] Agarwal N.P., Matthies M., Gür F.N., Osada K., Schmidt T.L. (2017). Block copolymer micellization as a protection strategy for DNA origami. Angew. Chem. Int. Ed..

[38] Ponnuswamy N., Bastings M.M.C., Nathwani B., Ryu J.H., Chou L.Y.T., Vinther M., Li W.A., Anastassacos F.M., Mooney D.J., Shih W.M. (2017). Oligolysine-based coating protects DNA nanostructures from low-salt denaturation and nuclease degradation. Nat. Commun..

[39] Ahmadi Y., Llano E.D., Barišić I. (2018). (Poly)cation-induced protection of conventional and wireframe DNA origami nanostructures. Nanoscale.

[40] Wang S.-T., Gray M.A., Xuan S., Lin Y., Byrnes J., Nguyen A.I., Todorova N., Stevens M.M., Bertozzi C.R., Zuckermann R.N. (2020). DNA origami protection and molecular interfacing through engineered sequence-defined peptoids. Proc. Natl. Acad. Sci. USA.

[41] Dobson C.M. (2003). Protein folding and misfolding. Nature.

[42] Riek R., Eisenberg D.S. (2016). The activities of amyloids from a structural perspective. Nature.

[43] Lou S., Wang X., Yu Z., Shi L. (2019). Peptide tectonics: Encoded structural complementarity dictates programmable self-assembly. Adv. Sci..

[44] Fletcher D.A., Mullins R.D. (2010). Cell mechanics and the cytoskeleton. Nature.

[45] Ke P.C., Zhou R., Serpell L.C., Riek R., Knowles T.P.J., Lashuel H.A., Gazit E., Hamley I.W., Davis T.P., Fändrich M. (2020). Half a century of amyloids: Past, present and future. Chem. Soc. Rev..

[46] Knowles T.P.J., Vendruscolo M., Dobson C.M. (2014). The amyloid state and its association with protein misfolding diseases. Nat. Rev. Mol. Cell Biol..

[47] Gremer L., Schölzel D., Schenk C., Reinartz E., Labahn J., Ravelli R.B.G., Tusche M., Lopez-Iglesias C., Hoyer W., Heise H. (2017). Fibril structure of amyloid-β(1–42) by cryo–electron microscopy. Science.

[48] Tayeb-Fligelman E., Tabachnikov O., Moshe A., Goldshmidt-Tran O., Sawaya M.R., Coquelle N., Colletier J.-P., Landau M. (2017). The cytotoxic *Staphylococcus aureus* PSMα3 reveals a cross-α amyloid-like fibril. Science.

[49] Engelberg Y., Landau M. (2020). The Human LL-37(17-29) antimicrobial peptide reveals a functional supramolecular structure. Nat. Commun..

[50] Channon K., Bromley E.H.C., Woolfson D.N. (2008). Synthetic biology through biomolecular design and engineering. Curr. Opin. Struct. Biol..

[51] Robson Marsden H., Kros A. (2010). Self-assembly of coiled coils in synthetic biology: Inspiration and progress. Angew. Chem. Int. Ed..

[52] Fletcher J.M., Boyle A.L., Bruning M., Bartlett G.J., Vincent T.L., Zaccai N.R., Armstrong C.T., Bromley E.H.C., Booth P.J., Brady R.L. (2012). A basis set of de novo coiled-coil peptide oligomers for rational protein design and synthetic biology. ACS Synth. Biol..

[53] Roy S., Caruthers M. (2013). Synthesis of DNA/RNA and their analogs via phosphoramidite and H-phosphonate chemistries. Molecules.

[54] Hao M., Qiao J., Qi H. (2020). Current and emerging methods for the synthesis of single-stranded DNA. Genes.

[55] Merrifield R.B. (1963). Solid phase peptide synthesis. I. the synthesis of a tetrapeptide. J. Am. Chem. Soc..

[56] Mäde V., Els-Heindl S., Beck-Sickinger A.G. (2014). Automated solid-phase peptide synthesis to obtain therapeutic peptides. Beilstein J. Org. Chem..

[57] Moss G.P., Smith P.A.S., Tavernier D. (1995). Glossary of class names of organic compounds and reactivity intermediates based on structure (IUPAC Recommendations 1995). Pure Appl. Chem..

[58] Ni R., Chau Y. (2014). Structural mimics of viruses through peptide/DNA co-assembly. J. Am. Chem. Soc..

[59] Ni R., Chau Y. (2017). Tuning the inter-nanofibril interaction to regulate the morphology and function of peptide/DNA co-assembled viral mimics. Angew. Chem. Int. Ed..

[60] Ni R., Chau Y. (2020). Nanoassembly of oligopeptides and DNA mimics the sequential disassembly of a spherical virus. Angew. Chem. Int. Ed..

[61] Lindsey J.S. (1991). Self-assembly in synthetic routes to molecular devices. biological principles and chemical perspectives. New J. Chem..

[62] Philp D., Stoddart J.F. (1996). Self-assembly in natural and unnatural systems. Angew. Chem. Int. Ed..

[63] Klug A. (1999). The tobacco mosaic virus particle: Structure and assembly. Philos. Trans. R. Soc. Lond. B. Biol. Sci..

[64] Zhou K., Ke Y., Wang Q. (2018). Selective *in situ* assembly of viral protein onto DNA origami. J. Am. Chem. Soc..

[65] Zhou K., Zhou Y., Pan V., Wang Q., Ke Y. (2020). Programming dynamic assembly of viral proteins with DNA origami. J. Am. Chem. Soc..

[66] Ruff Y., Moyer T., Newcomb C.J., Demeler B., Stupp S.I. (2013). Precision templating with DNA of a virus-like particle with peptide nanostructures. J. Am. Chem. Soc..

[67] Kang Z., Meng Q., Liu K. (2019). Peptide-based gene delivery vectors. J. Mater. Chem. B.

[68] Jiang T., Meyer T.A., Modlin C., Zuo X., Conticello V.P., Ke Y. (2017). Structurally ordered nanowire formation from co-assembly of DNA origami and collagen-mimetic peptides. J. Am. Chem. Soc..

[69] Jin J., Baker E.G., Wood C.W., Bath J., Woolfson D.N., Turberfield A.J. (2019). Peptide assembly directed and quantified using megadalton DNA nanostructures. ACS Nano.

[70] Buchberger A., Simmons C.R., Fahmi N.E., Freeman R., Stephanopoulos N. (2020). Hierarchical assembly of nucleic acid/coiled-coil peptide nanostructures. J. Am. Chem. Soc..

[71] Brizard A.M., van Esch J.H. (2009). Self-assembly approaches for the construction of cell architecture mimics. Soft Matter.

[72] Safont-Sempere M.M., Fernández G., Würthner F. (2011). Self-sorting phenomena in complex supramolecular systems. Chem. Rev..

[73] Draper E.R., Adams D.J. (2018). How should multicomponent supramolecular gels be characterised?. Chem. Soc. Rev..

[74] Kubota R., Nakamura K., Torigoe S., Hamachi I. (2020). The power of confocal laser scanning microscopy in supramolecular chemistry: In situ real-time imaging of stimuli-responsive multicomponent supramolecular hydrogels. ChemistryOpen.

[75] Udomprasert A., Bongiovanni M.N., Sha R., Sherman W.B., Wang T., Arora P.S., Canary J.W., Gras S.L., Seeman N.C. (2014). Amyloid fibrils nucleated and organized by DNA origami constructions. Nat. Nanotechnol..

[76] Higashi S.L., Shibata A., Kitamura Y., Hirosawa K.M., Suzuki K.G.N., Matsuura K., Ikeda M. (2019). Hybrid soft nanomaterials composed of DNA microspheres and supramolecular nanostructures of semi-artificial glycopeptides. Chem. Eur. J..

[77] Higashi S.L., Hirosawa K.M., Suzuki K.G.N., Matsuura K., Ikeda M. (2020). One-pot construction of multicomponent supramolecular materials comprising self-sorted supramolecular architectures of DNA and semi-artificial glycopeptides. ACS Appl. Bio Mater..

[78] Reches M., Gazit E. (2003). Casting metal nanowires within discrete self-assembled peptide nanotubes. Science.

[79] Görbitz C.H. (2001). Nanotube formation by hydrophobic dipeptides. Chem. Eur. J..

[80] Du X., Zhou J., Xu B. (2014). Supramolecular hydrogels made of basic biological building blocks. Chem. Asian J..

[81] Adler-Abramovich L., Gazit E. (2014). The physical properties of supramolecular peptide assemblies: From building block association to technological applications. Chem. Soc. Rev..

[82] Fleming S., Ulijn R.V. (2014). Design of nanostructures based on aromatic peptide amphiphiles. Chem. Soc. Rev..

[83] Tao K., Makam P., Aizen R., Gazit E. (2017). Self-assembling peptide semiconductors. Science.

[84] Tsuzuki T., Kabumoto M., Arakawa H., Ikeda M. (2017). The effect of carbohydrate structures on the hydrogelation ability and morphology of self-assembled structures of peptide–carbohydrate conjugates in water. Org. Biomol. Chem..

[85] Makam P., Gazit E. (2018). Minimalistic peptide supramolecular co-assembly: Expanding the conformational space for nanotechnology. Chem. Soc. Rev..

[86] Ikeda M. (2019). Stimuli-responsive supramolecular systems guided by chemical reactions. Polym. J..

[87] Draper E.R., Adams D.J. (2019). Controlling the assembly and properties of low-molecular-weight hydrogelators. Langmuir.

[88] Sugiura T., Kanada T., Mori D., Sakai H., Shibata A., Kitamura Y., Ikeda M. (2020). Chemical stimulus-responsive supramolecular hydrogel formation and shrinkage of a hydrazone-containing short peptide derivative. Soft Matter.

[89] Ohtomi T., Higashi S.L., Mori D., Shibata A., Kitamura Y., Ikeda M. (2020). Effect of side chain phenyl group on the self-assembled morphology of dipeptide hydrazides. Pept. Sci..

[90] Lampel A. (2020). Biology-inspired supramolecular peptide systems. Chem.

[91] Gour N., Kedracki D., Safir I., Ngo K.X., Vebert-Nardin C. (2012). Self-assembling DNA–peptide hybrids: Morphological consequences of oligonucleotide grafting to a pathogenic amyloid fibrils forming dipeptide. Chem. Commun..

[92] Gour N., Abraham J.N., Chami M., Castillo A., Verma S., Vebert-Nardin C. (2014). Label-free, optical sensing of the supramolecular assembly into fibrils of a ditryptophan–DNA hybrid. Chem. Commun..

[93] Nakamura Y., Yamada S., Nishikawa S., Matsuura K. (2017). DNA-modified artificial viral capsids self-assembled from DNA-conjugated β-annulus peptide. J. Pept. Sci..

[94] Matsuura K., Watanabe K., Matsuzaki T., Sakurai K., Kimizuka N. (2010). Self-assembled synthetic viral capsids from a 24-mer viral peptide fragment. Angew. Chem. Int. Ed..

[95] Nakamura Y., Inaba H., Matsuura K. (2019). Construction of artificial viral capsids encapsulating short DNAs via disulfide bonds and controlled release of DNAs by reduction. Chem. Lett..

[96] Matsuura K., Watanabe K., Matsushita Y., Kimizuka N. (2013). Guest-binding behavior of peptide nanocapsules self-assembled from viral peptide fragments. Polym. J..

[97] Matsuura K., Ueno G., Fujita S. (2015). Self-assembled artificial viral capsid decorated with gold nanoparticles. Polym. J..

[98] Fujita S., Matsuura K. (2016). Encapsulation of CdTe quantum dots into synthetic viral capsids. Chem. Lett..

[99] Matsuura K., Nakamura T., Watanabe K., Noguchi T., Minamihata K., Kamiya N., Kimizuka N. (2016). Self-assembly of Ni-NTA-modified β-annulus peptides into artificial viral capsids and encapsulation of His-tagged proteins. Org. Biomol. Chem..

[100] Matsuura K. (2018). Synthetic approaches to construct viral capsid-like spherical nanomaterials. Chem. Commun..

[101] Inaba H., Matsuura K. (2019). Peptide nanomaterials designed from natural supramolecular systems. Chem. Rec..

[102] Matsuura K., Ota J., Fujita S., Shiomi Y., Inaba H. (2020). Construction of ribonuclease-decorated artificial virus-like capsid by peptide self-assembly. J. Org. Chem..

[103] Matsuura K. (2020). Dressing up artificial viral capsids self-assembled from C-terminal-modified β-annulus peptides. Polym. J..

[104] Kye M., Lim Y.-B. (2016). Reciprocal self-assembly of peptide-DNA conjugates into a programmable sub-10-nm supramolecular deoxyribonucleoprotein. Angew. Chem. Int. Ed..

[105] Kye M., Lim Y. (2018). Synthesis and purification of self-assembling peptide-oligonucleotide conjugates by solid-phase peptide fragment condensation. J. Pept. Sci..

[106] Basavalingappa V., Bera S., Xue B., Azuri I., Tang Y., Tao K., Shimon L.J.W., Sawaya M.R., Kolusheva S., Eisenberg D.S. (2019). Mechanically rigid supramolecular assemblies formed from an Fmoc-guanine conjugated peptide nucleic acid. Nat. Commun..

[107] Basavalingappa V., Guterman T., Tang Y., Nir S., Lei J., Chakraborty P., Schnaider L., Reches M., Wei G., Gazit E. (2019). Expanding the functional scope of the Fmoc-diphenylalanine hydrogelator by introducing a rigidifying and chemically active urea backbone modification. Adv. Sci..

[108] Arakawa H., Takeda K., Higashi S.L., Shibata A., Kitamura Y., Ikeda M. (2020). Self-assembly and hydrogel formation ability of Fmoc-dipeptides comprising α-methyl-L-phenylalanine. Polym. J..

[109] Daly M.L., Gao Y., Freeman R. (2019). Encoding reversible hierarchical structures with supramolecular peptide–DNA materials. Bioconjug. Chem..

[110] Freeman R., Han M., Álvarez Z., Lewis J.A., Wester J.R., Stephanopoulos N., McClendon M.T., Lynsky C., Godbe J.M., Sangji H. (2018). Reversible self-assembly of superstructured networks. Science.

[111] Rha A.K., Das D., Taran O., Ke Y., Mehta A.K., Lynn D.G. (2020). Electrostatic complementarity drives amyloid/nucleic acid co-assembly. Angew. Chem. Int. Ed..

[112] Mou Y., Yu J.-Y., Wannier T.M., Guo C.-L., Mayo S.L. (2015). Computational design of co-assembling protein–DNA nanowires. Nature.

[113] Clarke N.D., Kissinger C.R., Desjarlais J., Gilliland G.L., Pabo C.O. (1994). Structural studies of the engrailed homeodomain. Protein Sci..

[114] Dirks R.M., Pierce N.A. (2004). Triggered amplification by hybridization chain reaction. Proc. Natl. Acad. Sci. USA.

[115] Fu T., Lyu Y., Liu H., Peng R., Zhang X., Ye M., Tan W. (2018). DNA-based dynamic reaction networks. Trends Biochem..

[116] McMillan J.R., Hayes O.G., Remis J.P., Mirkin C.A. (2018). Programming protein polymerization with DNA. J. Am. Chem. Soc..

[117] Figg C.A., Winegar P.H., Hayes O.G., Mirkin C.A. (2020). Controlling the DNA hybridization chain reaction. J. Am. Chem. Soc..

[118] Xu Y., Jiang S., Simmons C.R., Narayanan R.P., Zhang F., Aziz A.-M., Yan H., Stephanopoulos N. (2019). Tunable nanoscale cages from self-assembling DNA and protein building blocks. ACS Nano.

[119] Subramanian R.H., Smith S.J., Alberstein R.G., Bailey J.B., Zhang L., Cardone G., Suominen L., Chami M., Stahlberg H., Baker T.S. (2018). Self-assembly of a designed nucleoprotein architecture through multimodal interactions. ACS Cent. Sci..

[120] Brodin J.D., Ambroggio X.I., Tang C., Parent K.N., Baker T.S., Tezcan F.A. (2012). Metal-directed, chemically tunable assembly of one-, two- and three-dimensional crystalline protein arrays. Nat. Chem..

[121] Brodin J.D., Carr J.R., Sontz P.A., Tezcan F.A. (2014). Exceptionally stable, redox-active supramolecular protein assemblies with emergent properties. Proc. Natl. Acad. Sci. USA.

[122] Brodin J.D., Smith S.J., Carr J.R., Tezcan F.A. (2015). Designed, helical protein nanotubes with variable diameters from a single building block. J. Am. Chem. Soc..

[123] Keller A., Linko V. (2020). Challenges and perspectives of DNA nanostructures in biomedicine. Angew. Chem. Int. Ed..

[124] Ishihara K., Ohara S., Yamamoto H. (1996). 3,4,5-trifluorobenzeneboronic acid as an extremely active amidation catalyst. J. Org. Chem..

[125] Pattabiraman V.R., Bode J.W. (2011). Rethinking amide bond synthesis. Nature.

[126] Opie C.R., Noda H., Shibasaki M., Kumagai N. (2019). All non-carbon B_3_NO_2_ exotic heterocycles: Synthesis, dynamics, and catalysis. Chem. Eur. J..

[127] Liu Z., Noda H., Shibasaki M., Kumagai N. (2018). Catalytic oligopeptide synthesis. Org. Lett..

[128] Todorovic M., Perrin D.M. (2020). Recent developments in catalytic amide bond formation. Pept. Sci..

[129] Alberts B., Johnson A., Lewis J., Raff M., Roberts K., Walter P. (2002). Molecular Biology of the Cell.

[130] Suhanovsky M.M., Teschke C.M. (2015). Nature’s favorite building block: Deciphering folding and capsid assembly of proteins with the HK97-fold. Virology.

[131] Wu H., Fuxreiter M. (2016). The structure and dynamics of higher-order assemblies: Amyloids, signalosomes, and granules. Cell.

[132] Hoffman D.P., Shtengel G., Xu C.S., Campbell K.R., Freeman M., Wang L., Milkie D.E., Pasolli H.A., Iyer N., Bogovic J.A. (2020). Correlative three-dimensional super-resolution and block-face electron microscopy of whole vitreously frozen cells. Science.

[133] Shin Y., Brangwynne C.P. (2017). Liquid phase condensation in cell physiology and disease. Science.

[134] Kroschwald S., Alberti S. (2017). Gel or die: Phase separation as a survival strategy. Cell.

[135] Yoshizawa T., Nozawa R.-S., Jia T.Z., Saio T., Mori E. (2020). Biological phase separation: Cell biology meets biophysics. Biophys. Rev..

[136] Trivedi P., Palomba F., Niedzialkowska E., Digman M.A., Gratton E., Stukenberg P.T. (2019). The inner centromere is a biomolecular condensate scaffolded by the chromosomal passenger complex. Nat. Cell Biol..

[137] Herzel L., Ottoz D.S.M., Alpert T., Neugebauer K.M. (2017). Splicing and transcription touch base: Co-transcriptional spliceosome assembly and function. Nat. Rev. Mol. Cell Biol..

[138] Sanulli S., Trnka M.J., Dharmarajan V., Tibble R.W., Pascal B.D., Burlingame A.L., Griffin P.R., Gross J.D., Narlikar G.J. (2019). HP1 reshapes nucleosome core to promote phase separation of heterochromatin. Nature.

[139] Guo Y.E., Manteiga J.C., Henninger J.E., Sabari B.R., Dall’Agnese A., Hannett N.M., Spille J.-H., Afeyan L.K., Zamudio A.V., Shrinivas K. (2019). Pol II phosphorylation regulates a switch between transcriptional and splicing condensates. Nature.

[140] Du Y., Dong S. (2017). Nucleic acid biosensors: Recent advances and perspectives. Anal. Chem..

[141] Rubert Pérez C.M., Stephanopoulos N., Sur S., Lee S.S., Newcomb C., Stupp S.I. (2015). The powerful functions of peptide-based bioactive matrices for regenerative medicine. Ann. Biomed. Eng..

[142] Gelain F., Luo Z., Zhang S. (2020). Self-assembling peptide EAK16 and RADA16 nanofiber scaffold hydrogel. Chem. Rev..

[143] Armstrong J.P.K., Keane T.J., Roques A.C., Patrick P.S., Mooney C.M., Kuan W.-L., Pisupati V., Oreffo R.O.C., Stuckey D.J., Watt F.M. (2020). A blueprint for translational regenerative medicine. Sci. Transl. Med..

[144] Cutler J.I., Auyeung E., Mirkin C.A. (2012). Spherical nucleic acids. J. Am. Chem. Soc..

[145] Tan X., Jia F., Wang P., Zhang K. (2020). Nucleic acid-based drug delivery strategies. J. Control. Release.

[146] Jiang Z., Thayumanavan S. (2020). Noncationic material design for nucleic acid delivery. Adv. Therap..

[147] Roberts T.C., Langer R., Wood M.J.A. (2020). Advances in oligonucleotide drug delivery. Nat. Rev. Drug Discov..

